# Dysregulation of Astrocyte Ion Homeostasis and Its Relevance for Stroke-Induced Brain Damage

**DOI:** 10.3390/ijms22115679

**Published:** 2021-05-26

**Authors:** Michel J.A.M. van Putten, Christoph Fahlke, Karl W. Kafitz, Jeannette Hofmeijer, Christine R. Rose

**Affiliations:** 1 Department of Clinical Neurophysiology, University of Twente, 7522 NB Enschede, The Netherlands; m.j.a.m.vanputten@utwente.nl (M.J.A.M.v.P.); j.hofmeijer@utwente.nl (J.H.); 2 Institut für Biologische Informationsprozesse, Molekular-und Zellphysiologie (IBI-1), Forschungszentrum Jülich, 52425 Jülich, Germany; c.fahlke@fz-juelich.de; 3 Institute of Neurobiology, Heinrich Heine University Düsseldorf, 40225 Düsseldorf, Germany; kafitz@hhu.de

**Keywords:** stroke core, penumbra, sodium, potassium, pH, chloride, glutamate transport, edema, cell swelling

## Abstract

Ischemic stroke is a leading cause of mortality and chronic disability. Either recovery or progression towards irreversible failure of neurons and astrocytes occurs within minutes to days, depending on remaining perfusion levels. Initial damage arises from energy depletion resulting in a failure to maintain homeostasis and ion gradients between extra- and intracellular spaces. Astrocytes play a key role in these processes and are thus central players in the dynamics towards recovery or progression of stroke-induced brain damage. Here, we present a synopsis of the pivotal functions of astrocytes at the tripartite synapse, which form the basis of physiological brain functioning. We summarize the evidence of astrocytic failure and its consequences under ischemic conditions. Special emphasis is put on the homeostasis and stroke-induced dysregulation of the major monovalent ions, namely Na^+^, K^+^, H^+^, and Cl^−^, and their involvement in maintenance of cellular volume and generation of cerebral edema.

## 1. Introduction

Cerebral ischemia is a pathological condition in which blood flow to the brain is insufficient to meet the brain’s high metabolic demands. Ischemia may be focal, if a brain artery is occluded, leading to ischemic stroke or brain infarction. Cerebral ischemia may also affect the whole brain, for example during cardiac arrest. Reversible loss of cerebral functioning and irreversible damage come sequentially on time scales of minutes to days, depending on the remaining perfusion level and metabolic demand of the brain region at stake [1,2]. The only treatment of proven benefit is rapid reperfusion with 6–24 h after symptom onset. The sooner reperfusion is achieved, the better a patient’s prognosis, reflecting the high vulnerability of the brain to hypoperfusion and hypoxia [3,4].

**Figure 1:**
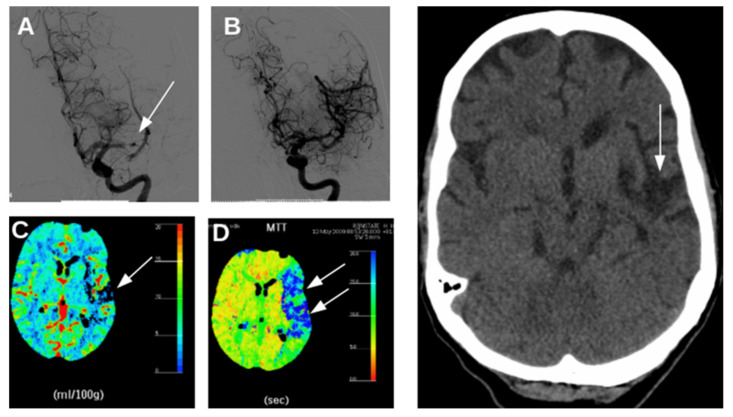
Radiology images from a patient with an acute ischemic stroke caused by occlusion of the left middle cerebral artery. (**A**): Digital subtraction angiography at 3 h after symptom onset obtained by intra-arterial administration of contrast agent, conforming middle cerebral artery occlusion. (**B**): revascularization after mechanical thrombectomy. (**C**): CT-perfusion maps, showing reduced cerebral blood volume (CBV) in a small part of the left hemisphere reflecting the infarct core (arrow) and (**D**) prolonged mean transit time (MTT) in a large part of the left hemisphere (blue tones in right panel, white arrows) reflecting hypoperfusion in the penumbra. Right: CT scan of the head 3 months after thrombectomy, showing a remaining lesion at the side of the previous infarct core (arrow), but recovery of brain areas that were part of the penumbra. The patient recovered completely.

In ischemic stroke, perfusion levels may range from <5 mL/100 g/min in the core of the affected vascular territory to >35 mL/100 g/min in peripheral regions, through inflow of blood from surrounding arteries [5]. In the core, energy supply is classically insufficient to preserve ion gradients across the plasma membrane, with depolarization, Ca^2+^ influx, cell swelling, and irreversible cell damage within minutes [6]. In the surrounding regions, the so-called ischemic penumbra, synaptic activity may fail, but neurons and astrocytes initially remain structural intact and viable [7]. If blood flow is restored in time, cerebral dysfunction is, in principle, reversible (Figure 1). However, if oxygen and glucose are not resupplied, there will be progression towards irreversible failure of neurons and astrocytes on timescales of hours to days [8,9].

Neurophysiological processes that contribute to recovery or ongoing damage are only partially understood. Increased concentrations of excitatory amino acids (mainly glutamate) in the extracellular space (ECS) will trigger excessive influx of cations through ionotropic glutamate receptors, namely NMDA, AMPA, and KA receptors, into the cell [10]. This is termed ‘excitotoxicity’, since the resulting prolonged depolarization and Ca^2+^ load will lead to activation of enzymes that degrade membranes, proteins, and nucleic acids [11,12]. ‘Spreading depolarization’ refers to propagation of neuronal and glial depolarization across the brain, resulting from diffusion of excitatory substances such as glutamate and K^+^ through the ECS [13]. The strong cellular depolarization leads to suppression of all spontaneous or evoked electrical activity, a phenomenon referred to as ‘spreading depression’ [14,15].

Excitotoxicity and spreading depolarization are associated with transitions from reversible to irreversible neuronal damage in penumbral areas surrounding the infarct core. In peripheral regions of the penumbra, where remaining blood flow levels are presumably higher, persistent synaptic failure may result in lasting brain damage in the absence of membrane depolarization [16]. Of note, however, many studies discourage the overly simplified discrimination between infarct core and penumbra [17], and a vast heterogeneity with regard to cellular and molecular dynamics in core as well as penumbra has been demonstrated [18].

Many of the biochemical and electrophysiological processes contributing to neuronal damage during cerebral ischemia, such as glutamate release, depolarization, and synaptic modulations, are key to normal brain functioning and not harmful under physiological conditions. Damage comes from energy depletion impeding restoration of distorted homeostasis and ion gradients. Astrocytes play a key role in keeping brain homeostasis by ion buffering [19], synthesis and uptake of neurotransmitters [20], water transport [21], energy distribution [22], and immunomodulation [23,24]. For these reasons, astrocytes have been proposed as high potential candidate targets for neuroprotective treatments to prevent or delay transitions from reversible to irreversible neuronal damage in the penumbra [25,26,27,28,29]. In this context, many of the previous studies and reviews have focused on astrocytes’ roles in immune responses, angiogenesis, Ca^2+^ buffering as potential targets to prevent cell death [30,31] and/or emphasized the relevance of astrocyte activation upon stroke [32]. Here, we review the pivotal role of astrocytes at the tripartite synapse with a focus on energy supply and monovalent ion homeostasis, which form the basis of both physiological neuronal cell function and dysfunction under ischemic conditions.

## 2. Energy Dependence of the Tripartite Synapse

### 2.1. Energy Needs of Neurons and Astrocytes

The brain is a very hungry organ. In humans at rest, the CNS uses more than 20% of the entire O_2_ consumption and its energy needs relative to its weigh thus exceed those of all other organs [33,34]. Because ATP is mainly generated by mitochondrial respiration, the brain strictly depends on the continuous supply of oxygen and glucose, making it highly vulnerable to conditions of reduced blood flow.

**Figure 2:**
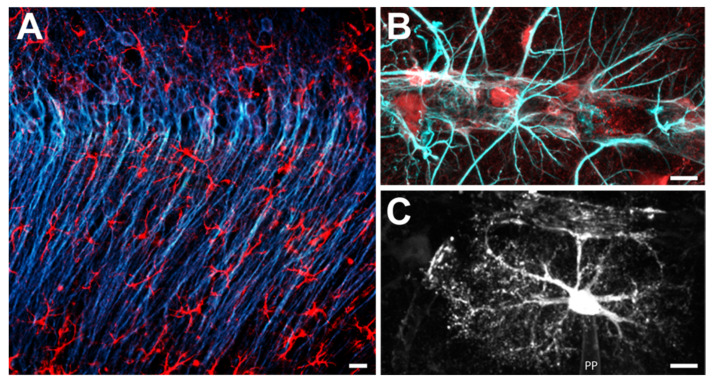
Astrocytes in the CA1 region of the mouse hippocampal formation. (**A**) Double-immunohistochemical labelling for the neuronal marker MAP-2 (blue) and for the astroglial marker GFAP (red). (**B**) Double-immunohistochemical labelling for the astroglial markers S-100β (red) and GFAP (light blue) along a blood vessel. Note the many processes that terminate at or parallel the blood vessel. (**C**) Astrocyte, dye-filled via a patch pipette (PP). Scale bars: (**A**) 5 μm; (**B**,**C**) 20 μm.

Earlier estimates indicate that 25–40% of the cellular ATP are used for housekeeping processes not directly related to signalling such as turnover of microtubules, treadmilling of the actin cytoskeleton or lipid turnover [34,35]. The major share of ATP, however, is consumed by ion pumps embedded into plasma membranes and membranes of intracellular organelles responsible for the maintenance or re-establishment of ion gradients between different compartments [36,37,38]. Electrical signalling by neurons relies on the flux of ions and erodes these ion gradients. The subsequent restoration of ion gradients accounts for the majority of activity-related ATP consumption in the brain [34,39,40]. Astrocytes, which are in close spatial and functional relationships with neurons (Figure 2), are essentially electrically silent, and require only 5–10% of the total energy budget of neural activity [39,40].

**Figure 3:**
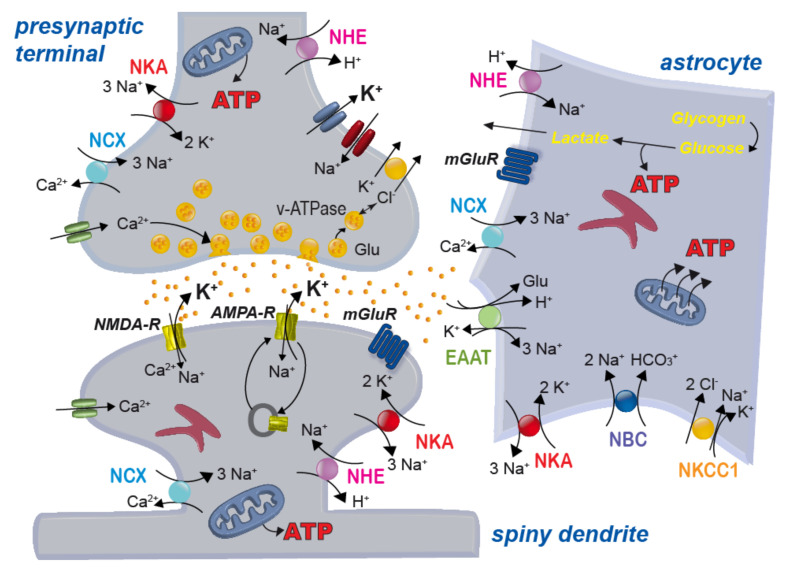
Scheme of a glutamatergic synapse and main ion transport mechanisms across the plasma membrane of neurons and astrocytes. NHE: sodium/proton exchanger; NKA: sodium/potassium-ATPase; NCX: sodium/calcium exchanger; NBC: sodium-bicarbonate-cotransporter; NKCC1: sodium-potassium-2 chloride cotransporter 1; EAAT: excitatory amino acid transporter; NMDA/AMPA-R: NMDA/AMPA-receptor; mGluR: metabotropic glutamate receptor; v-ATPase, vesicular ATPase; Glu: glutamate.

ATP is primarily used to enable the activity of ion pumps, most notably the Na^+^/K^+^-ATPase (NKA) [41,42], the Ca^2+^-ATPases of the plasma membrane (PCMA) and endoplasmic reticulum (SERCA) [34,43] and the H^+^-ATPase of presynaptic vesicles (v-ATPase) [44,45,46] (see Figure 3). Among these pumps, the NKA is the major cellular energy consumer. The NKA establishes a low intracellular Na^+^ concentration by exporting 3 Na^+^ against 2 K^+^ [41,42,47,48], thereby generating the main driving forces for electrical signalling.

Besides its pivotal role for fast electrical signalling based on Na^+^ and/or K^+^ flux through ion channels, the NKA enables secondary-active transport, including the Na^+^/Ca^2+^ exchanger (NCX) or the Na^+^/H^+^ exchanger (NHE) [37,49] (see Figure 3). While these transporters do not consume ATP, a reduction in NKA activity from ATP depletion decreases Na^+^ gradients, and indirectly affects their function. This not only may cause a reduction in transport rate. Depending on the severity of ATP shortage and the resulting breakdown of ion gradients, transporters may even reverse–with often detrimental consequences as is e.g., the case for Na^+^-dependent glutamate transport [36,37,50,51], see also below).

### 2.2. Neuro-Metabolic Coupling between Neurons and Astrocytes

Chemical synapses of the vertebrate brain are usually illustrated as two-compartment systems consisting of a presynaptic terminal and a postsynaptic neuron. As a matter of fact, this is a rather simplified view. Neurons in the brain are surrounded by a narrow ECS that largely restricts diffusion. Recent super-resolution imaging studies in live tissue showed that the widths of the interstitial spaces, as determined in organotypic tissue slices of the CA1 area of the mouse hippocampus, ranges from 50 nm to over 1 μm (median 270 nm) and can change dynamically with neuronal activity [52]. Moreover, neurons do not have direct access to blood vessels. Still and despite these apparent restrictions, pre- and postsynaptic neuronal compartments-which are the major consumers of cellular ATP-need rapid and sufficient access to energy sources to cope with periods of high activity. Neurons, however, do not have any significant stores for metabolites that could quickly be fed into glycolysis or oxidative phosphorylation. Instead, astrocytes that contact synapses with their fine perisynaptic processes [53,54,55], are perfectly suited to help neurons out; exactly those cells that have an apparently low energy requirement on their own and store considerable amounts of glycogen [56,57]. In addition, perivascular endfeet of astrocytes essentially fully cover blood vessels, enabling direct uptake of glucose and O_2_ from the blood [58] (Figure 2).

There is overwhelming evidence for a key role of astrocytic glycogen in the healthy and diseased brain [59,60]. Moreover, direct neuro-metabolic coupling between neurons and astrocytes supports periods of high neuronal energy demand. Activity of neurons results in increased uptake of glucose from the blood by neighbouring astrocytes, accompanied by increased glycolysis and breakdown of glycogen [59,61]. Stimulation of astrocytic glycolysis by neuronal activity involves several mechanisms. A prominent role is played by the astrocytic sodium-bicarbonate-cotransporter (NBCe1) (Figure 3). NBCe1 is activated by K^+^ released from active neurons, resulting in an intracellular alkalinization of astrocytes enhancing glycolysis [62]. Moreover, astrocytic glycolysis is stimulated by increases in intracellular Na^+^ which accompany neuronal activity [63,64]. Lactate produced through glycolysis is then released from astrocytes and taken up by neurons [61,65,66] (Figure 3). Analogous neuro-metabolic coupling was proposed for white matter, with oligodendrocytes supporting active neurons with lactate [67,68]. There is still substantial debate about the existence of the astrocyte-neuron-lactate shuttle in vivo ([62,69,70,71,72,73]), so further work is clearly needed to solve this issue. Activation of astrocytes’ metabolism by neuronal activity, however, is undebated.

### 2.3. Housekeeping by Astrocytes and the Tripartite Synapse

As mentioned above, neurons in the brain are tightly packed and surrounded by a narrow ECS [52]. The properties of the ECS need to be tightly controlled which includes the regulation of the concentrations of ions and neurotransmitters. Astrocytes play centre stage in managing these tasks [74]. Their processes reach close to synapses, hindering diffusion in the narrow ECS [75]. In some brain areas, astrocytes even completely wrap around individual synapses, preventing spill-over of glutamate [76]. One of the first processes of neuron-glia interaction described was the ability of astrocytes to sense and control increases in extracellular K^+^ concentration [77]. Even with prolonged neuronal activity (and accompanying release of K^+^), astrocytes keep extracellular K^+^ concentrations below a ceiling level of 10–12 mM [78,79]. This process, which is central to prevent uncontrolled depolarization of neurons [80], is mainly mediated by uptake of K^+^ by the astrocyte NKA [81,82]. As a consequence, elevation of extracellular K^+^ results in a decrease in the intracellular Na^+^ concentration of astrocytes [83,84,85]. In addition, uptake of K^+^ by astrocytes involves Kir4.1 channels [86]. The sodium-potassium-2 chloride-cotransporter 1 (NKCC1) (Figure 3) mediates astrocytic K^+^ uptake under conditions of strongly elevated extracellular K^+^ [81]. Once taken up, K^+^ can pass through gap junctions and this process, called spatial buffering, helps to redistribute K^+^ in the astrocyte network [86].

Astrocytes also control the extracellular concentration of the major neurotransmitters. Among the most essential tasks is the uptake of glutamate by high-affinity excitatory amino acid transporters (EAAT) (Figure 3), namely the glutamate aspartate transporter 1 (GLAST/EAAT1) and glutamate transporter 1 (GLT1/EAAT2). Glutamate uptake by astrocytes is absolutely critical for brain function since it protects neurons from glutamate-mediated overexcitation (termed excitotoxicity) [20,87,88,89]. Once taken up, glutamate can be converted to α-ketoglutarate and be fed into the TCA cycle [90,91]. Alternatively, it is converted to glutamine, which is exported into the ECS and again taken up by neurons to serve as a precursor for synthesis of glutamate or GABA [87].

In addition to fulfilling homeostatic roles, astrocytes actively contribute to signaling at synapses. Such active interaction can be triggered by astrocytic Ca^2+^ transients and involves their release of neuroactive substances, such as ATP and glutamate, which bind to receptors on surrounding neurons. At glutamatergic synapses, astrocyte Ca^2+^-signaling is e.g., induced upon activation of metabotropic glutamate receptors (mGluR5), at least in the juvenile brain [92,93]. Other pathways for the generation of astrocyte Ca^2+^ transients include reverse NCX, TRPA1 channels or P2Y1 receptors [94]. In consideration of this active role, the term “tripartite” synapse was introduced [95,96,97]. It acknowledges the fact that communication, information transfer and plasticity of chemical synapses involve more than the two neuronal cells, but include a third, astrocytic, compartment. As for the astrocyte-neuron-lactate shuttle, this topic is still partly questioned in the field [98,99]. There are many excellent reviews to which the reader is referred here [54,55,94,100,101].

## 3. Relevance of Astrocytic Cation Homeostasis

### 3.1. Sodium Homeostasis

Astrocytes maintain a low intracellular Na^+^ concentration of 10–15 mM at rest, which is achieved by the action of the NKA, the only relevant export pathway for Na^+^ across the plasma membrane [49,102]. Astrocytes predominately express α2/β2 subunits of the NKA, which exhibit a K_D_ of ~4 mM for extracellular K^+^, making it ideally suited to take up K^+^ released by active neurons [103]. NKA thus not only determines intracellular Na^+^ and K^+^ of astrocytes, but also contributes to regulation of extracellular K^+^.

Astrocyte Na^+^ homeostasis is deeply intermingled with the regulation of all other major ions through secondary and tertiary Na^+^-dependent transporters. It is thereby directly coupled to homeostasis of intracellular Ca^2+^ (through NCX) and intracellular pH (through NHE and NBCe1) [49,51,102]. Notably, NCX is readily reversible, and increases in astrocytic Na^+^ may turn Ca^2+^ export into import, resulting in intracellular Ca^2+^ signaling [51]. Another transporter that works close to its reversal potential is the NBCe1 [104,105]. Besides regulating ion homeostasis, the Na^+^ gradient enables the uptake of transmitters from the ECS trough the aforementioned Na^+^-dependent glutamate transporters EAAT1 and EAAT2 as well as through transporters for GABA [106,107].

While keeping astrocytic Na^+^ low is critical for the function of an entire battery of Na^+^-dependent transporters, it has become clear that activation of the latter in response to neuronal activity also results in well-detectable transient increases in intracellular Na^+^ [74,102,108,109]. Activity-induced Na^+^ transients are largest in fine astrocytic processes close to activated synapses, but–depending on strength and pattern of activity-may also occur in somata or perivascular endfeet [110]. Major Na^+^-loaders for astrocytes are the EAATs, which can increase astrocytic Na^+^ by up to 10 mM with short burst stimulation of glutamatergic fibers [111,112,113]. In addition, Na^+^-permeable ion channels, among them AMPA or NMDA receptors expressed by some types of astrocytes may contribute to Na^+^ signals [74,111,114]. Subsequent export of Na^+^ is primarily mediated by the NKA, but a (Na^+^-driven) reversal of the NCX may assist the NKA in exporting Na^+^ [51,109]. Upon induction of local influx, Na^+^ rapidly diffuses to non-stimulated areas, thereby readily passing gap junctions and invading neighboring cells [110,115,116,117]. Because lateral diffusion will reduce local activation of the NKA, this re-distribution of Na^+^ is likely to result in a distribution of the metabolic load within the astrocytic syncytium.

### 3.2. Sodium Dysregulation and Its Consequences under Ischemic Conditions

Intracellular Na^+^ homeostasis is directly coupled to the NKA and inhibition of NKA results in an immediate rise in Na^+^ in cultured astrocytes even under resting conditions [84,118]. Because NKA requires ATP to function, intracellular Na^+^ homeostasis is also strictly dependent on an intact energy metabolism [36,119]. An increase in intracellular Na^+^ and a resulting breakdown of the Na^+^ gradient are thus immediate and primary consequences of energy deprivation arising from failure of cellular ATP production [36,37,109,120].

Na^+^ loading in response to metabolic inhibition can be easily demonstrated in resting cultured astrocytes. Chemical ischemia induced by blockers of glycolysis and oxidative respiration results in an instant rise in Na^+^ in cultured spinal cord and cortical/forebrain astrocytes as well as in C6 glioma cells [118,121,122,123]. The degree of Na^+^ dysbalance is dependent on the specific preparation as well as on the severity and duration of energy deprivation. This is also true for the intact tissue. In acute slices of the mouse neocortex, perfusion with metabolic inhibitors for 2 min transiently increased Na^+^ by ~8 mM and a 5-min perfusion by 30 mM [124]. This protocol mimicked transient Na^+^ accumulations by 10–20 mM that were observed in the ischemic penumbra of the mouse neocortex in vivo during peri-infarct depolarisations (PIDs) [124]. While in vivo data are not available from ischemic core regions, astrocytic Na^+^ loading is certainly substantially higher in the core [37,50].

The consequences of an increase in intracellular Na^+^ under ischemic conditions are manifold. Elevating astrocyte Na^+^ activates glycolysis and breakdown of glycogen, and astrocytes might be able to produce lactate for a limited period of time by anaerobic glycolysis [62,125,126,127]. It is unclear if an uptake of astrocyte-derived lactate by neurons would be solely beneficial under hypoxic conditions, because it is accompanied by uptake of protons, exacerbating cellular acidosis and thereby possibly exacerbating neuronal damage [128].

The increase in intracellular Na^+^ during energy deprivation is also accompanied by a reduction of the astrocytic membrane potential [129]. In astrocytes of acute hippocampal slices, an initial slow rise, followed by a faster and more prominent depolarization to a plateau was observed in response to oxygen-glucose deprivation for 30 min [130]. In addition to influx of Na^+^, an increase in extracellular K^+^ was identified as a major cause for the astrocyte depolarization [130,131]. Its degree again depends on the preparation, model system and on the severity/duration of ATP depletion. Notably, both membrane depolarization and Na^+^ loading not only affect astrocytes themselves, but will severely impact neuron-glia interaction, and thereby, synaptic transmission. Considering the above-described roles of astrocytes in ionic homeostasis and re-uptake of transmitters in which Na^+^ homeostasis is involved, the resulting functional effects are significant, if not even dramatic.

Disturbance of astrocytic metabolism by sodium fluoroacetate (NaFAc), which selectively inhibits the enzyme aconitase in astrocytes [132], not only elevated intracellular Na^+^, but also caused a significant reduction in their K^+^ uptake via the NKA [83]. As a result, extracellular K^+^ transients accompanying burst firing of neurons were increased in amplitude and duration. In addition, the astrocyte’s capacity to take up glutamate was reduced [83]. This was accompanied by a prolongation of neuronal burst firing, indicating increased neuronal excitability [83].

While in the above-mentioned studies, Na^+^ increases were moderate (10–20 mM), and thus in the same range as those observed during PIDs in vivo [124], the situation will differ in the ischemic core. Here, strong cellular depolarization together with a presumed substantial increase in intracellular Na^+^ will likely cause reverse operation of glutamate transporters [133]. Such reversal will drive accumulation of glutamate in the ECS, causing drive excitotoxicity and cell death [50,125,133,134,135,136]. Importantly, cell death in response to extracellular glutamate accumulation not only relates to neurons, but has also been described for neocortical astrocytes and oligodendrocytes [137].

In addition to transport reversal, ischemic conditions may result in Ca^2+^-dependent vesicular release of glutamate from astrocytes [97,138,139]. In mice subjected to a transient MCAO, spreading depolarisations travelling across the cortical penumbra are accompanied by transient Ca^2+^-increases in astrocytes, mediated both by influx through TRPV4 channels and by reverse NCX [124,140]. Na^+^-driven reversal of NCX could thus augment such vesicular release of glutamate from astrocytes [51,108,141]. Moreover, reverse NCX-mediated Ca^2+^-loading might directly harm astrocytes inducing mitochondrial Ca^2+^ overload and Ca^2+^-dependent cell death [141,142,143].

### 3.3. Potassium

Studies providing direct experimental data on intracellular K^+^ concentrations in astrocytes are rare. The main reason is the lack of reliable indicators for K^+^ imaging, necessitating the use of (invasive) ion-selective microelectrodes (e.g., [144,145]). Mostly based on such invasive studies, it has been established that astrocytic K^+^ concentration is about 10-fold higher than that of Na^+^ [146]. This was confirmed by a more recent imaging approach employing the chemical indicator APG-1, in which a K^+^ concentration of about 130 mM was determined for cultured astrocytes at rest [147]. Astrocyte K^+^ is mainly governed by the NKA [146]. Another transporter coupling transport of both ions is NKCC1, albeit mediating influx of K^+^ into astrocytes [148]. Finally, Na^+^-dependent glutamate transporters (EAATs) export 1 K^+^ per transport cycle [20]. Astrocytes also show substantial expression of different types of voltage-dependent plasma membrane K^+^ channels, KIR4.1 being the most prominent one [146,149].

Under physiological conditions, astrocytes take up K^+^ released from active neurons by both channel- and transport-mediated mechanisms and thereby regulate neuronal excitability [37,82,103,146]. Exogenous application of glutamate, in contrast, resulted in a decline in astrocytic K^+^, most likely as a consequence of activation of EAATs [147]. Movement of K^+^ across the plasma membrane mediated by the above-mentioned mechanisms is opposed to that of Na^+^ (e.g. [83]), again exemplifying the close interrelation between both ions.

There is a wealth of data and investigations demonstrating that energy restriction and accompanying spreading depolarization events are accompanied by a failure of extracellular K^+^-homeostasis in which astrocytes are critically involved [134,150,151,152]. A recent study also proposed an involvement of microglia in extracellular K^+^-homeostasis during spreading depolarisations [153]. In most models of prolonged energy failure, extracellular K^+^ initially rises slowly up to a ceiling level of about 10–12 mM, upon which there is a second steep rise to a plateau of more than 50–60 mM [125,129,134]. Failure of astrocyte NKA is a major reason for the breakdown of the K^+^ ceiling level, ultimately also leading to a loss of K^+^ from astrocytes [131]. Loss of astrocytic K^+^ will be further promoted by ATP-sensitive K^+^-channels (K_ATP_), which open upon a decline in intracellular ATP [24]. Knockout of Kir6.1, the pore-forming unit of astrocytic K_ATP_, aggravated ischemic lesions und edema in the mouse brain [154].

K^+^ efflux from astrocytes under ischemic conditions aggravates the accumulation of extracellular K^+^ induced by its release from neurons. The substantial elevation of extracellular K^+^ in turn activates NKCC1, driving import of Na^+^, K^+^ and Cl^−^ and resulting in substantial cellular swelling of astrocytes [155,156]. Inhibition of NKCC1 significantly reduces infarct volumes, making this transporter highly relevant in the generation of ischemic damage in the brain [143,157] (see also below).

### 3.4. pH

Astrocytes maintain an intracellular pH in the range of 7.2 (corresponding to a proton concentration of 63 nM) at rest [158,159]. Like Ca^2+^, protons are heavily buffered in the cytosol, the HCO_3_^-^/CO_2_ system being the major mechanism of buffering [158]. Astrocyte pH is higher than expected from a passive distribution of protons, so export of acid equivalents into the ECS is required to counteract intracellular acidification [160]. Two Na^+^-dependent transporters are involved in the regulation of intracellular pH [159]. One is the NHE, more specifically the isoform NHE1, which mediates the electroneutral exchange of protons with Na^+^ [143]. The second transporter is NBCe1, which transports HCO_3_^−^ along with 2 Na^+^ across the membrane. NBCe1 operates close to its reversal potential and can thereby serve as an acid extruder, acidifying the ECS, an as well as an acid loader, removing acid equivalents from the ECS [158,160]. The latter dampens extracellular acidifications accompanying neuronal activity [161].

Inhibition of metabolism and ischemic conditions cause a rapid drop in extracellular pH, which–depending on the depth and duration of ischemia-may reach values between 6.5 and 6.9 [120,162,163,164]. Waves of spreading depolarization result in transient, but long-lasting acidification of the ischemic cortex of rats [165]. Acidosis accompanying ischemia is a harmful event, reducing astrocyte glutamate uptake [166] and exacerbating cytotoxic edema and cellular damage [167]. When exposed to a low extracellular pH, astrocytes are unable to maintain their pH above 7, but acidify and may even undergo cell death [159,162,168]. Moreover, inhibition of anaerobic metabolism moreover results in an intracellular acidification of astrocytes as a consequence of glycogen breakdown and glycolytic production of lactate [169]. The subsequent strong activation of NHE1 ameliorates intracellular acidosis–while at the same time serving as a strong pathway for Na^+^ loading, promoting cellular swelling and cell death [123,143,170,171].

In contrast to NHE1, NBCe1 has been reported to serve a predominately neuroprotective role [172]. In astrocytes derived from human-induced pluripotent stem cells, pharmacological inhibition of NBCe1 resulted in increased cell death in response to exposure to ischemic conditions [163]. Again, while inwardly-directed NBCe1 will dampen the astrocytic acidification, Na^+^ influx will be aggravated–with all the negative consequences outlined above. NBCe1 has also been implemented in astrocyte swelling [173], which, if this process is operative under ischemic conditions, will most likely promote tissue damage.

Notably, a recent study reported a direct causal relationship between glial pH and glutamate-related excitoxicity in the cerebellum [174]. Induction of proton influx into Bergmann glial cells through activation of channelrhodopsin-2 resulted in a decrease in intracellular pH, triggering a release of glutamate from the glial cells. Conversely, optogenetic induction of proton export from Bergmann glial cells reduced their release of glutamate and significantly reduced ischemic brain damage in vivo [174]. These results suggested that pH-dependent glial glutamate release contributes to excitotoxic cell death. Glial acidosis thus emerged as one of the mechanisms inducing neuronal damage in the ischemic brain [175].

## 4. Relevance of Astrocytic Chloride Homeostasis

### 4.1. Regulation of Intracellular Chloride Levels

Chloride (Cl^−^) is the most abundant anion in the human body, and every cell of our body expresses multiple Cl^−^ channels and transporters to adjust Cl^−^ concentrations in the cytoplasm and in cell organelles. Water flux out of the cell or into the cell is always linked to Cl^−^ fluxes in the same direction, making the intracellular Cl^−^ concentration ([Cl^−^]_i_) a main determinant of cell swelling and regulatory volume changes [176,177]. Volume regulation and thus Cl^−^ homeostasis are especially important for astrocytes, since they undergo fast changes in cell volume when exposed to osmotic gradients [178,179,180,181]. Moreover, changes in osmotically active solute concentrations are intimately associated with glial key functions.

Despite its high physiological importance, [Cl^−^]_i_ has only been determined for few cell types, and mechanisms underlying intracellular Cl^−^ homeostasis remain insufficiently understood [182,183]. So far, glial [Cl^−^]_i_ has been experimentally addressed in cultured astrocytes with results varying between of 29–46 mM [184,185,186,187,188,189]. In acute mouse brain tissue slices, fluorescence lifetime imaging microscopy (FLIM) with the Cl^−^-sensitive dye MQAE [190,191,192] provided a [Cl^−^]_i_ of 34 mM for cerebellar Bergmann glia cells [192,193]. These high values predict a Cl^−^ reversal potential positive to the resting potential in glial cells. Astrocytes express GABA_A_ receptors (GABA_A_Rs) [194,195], anion-selective channels that open upon binding of GABA to an extracellular binding site. In cultured astrocytes and as well as in astrocytes in acute brain slices, electrophysiological experiments demonstrated astrocyte depolarization upon activation of GABA_A_ receptors [196,197], in agreement with astrocytic [Cl^−^]_i_ significantly higher than expected for a passive distribution.

A combination of specific inhibitors and genetically altered animals was used to identify anion transporters responsible for setting [Cl^−^]_i_ in Bergmann glial cells [193]. Blocking NKCC1 decreased [Cl^−^]_i_. Genetic ablation as well as pharmacological inhibition of EAATs and block of potassium-chloride cotransporters (KCC) resulted in its rise. [Cl^−^]_i_ of Bergmann glia is thus in dynamic equilibrium between transport processes that accumulate Cl^−^, such as NKCC1, and transporters or channels that move Cl^−^ out of the cells, such as KCC1 and KCC3 or EAAT anion channels [193]. It is safe to assume that astrocytes exhibit similar mechanisms of intracellular Cl^−^ homeostasis.

The importance of cation Cl^−^ cotransporters for cellular Cl^−^ homeostasis has been known for many years [198,199,200,201]. However, the involvement of glutamate transporters came as surprise. EAATs are not only secondary-active glutamate transporters, but also anion channels [202,203,204], with anion channel opening being closely linked to conformational changes underlying glutamate transport [205,206,207]. A role in regulating [Cl^−^]_i_ in glia was one of the first cellular functions unambiguously assigned to EAAT anion channels. [Cl^−^]_i_ was studied at different developmental stages, revealing a glial Cl^−^ switch [193], which is reminiscent of the neuronal Cl^−^ switch [182]. In very young animals (P5–6), the Bergmann glial intracellular Cl^−^ concentration was >50 mM, and dropped to the adult value of 34 mM around P14. This Cl^−^ switch coincides with the age-related upregulation of EAAT1/GLAST and EAAT2/GLT-1 [208], and block of both glial glutamate transporters increases [Cl^−^]_i_ in adult Bergmann glia to juvenile levels. Changes in glial Cl^−^ homeostasis caused by mutations in *SLC1A3* that encodes EAAT1 play a central role in the disease pathogenesis of certain forms of episodic ataxia, an inherited neurological conditions. A severe form of episodic ataxia characterized by ataxia and epilepsy was associated with a proline by arginine substitution at position 290 in EAAT1 [209]. This mutation increased the activity of EAAT1 anion channels, and enhanced mutant EAAT1-mediated Cl^−^ efflux between P8 and P13 triggers Bergmann glia shrinking and apoptosis in an animal model of this disease [210,211].

**Figure 4:**
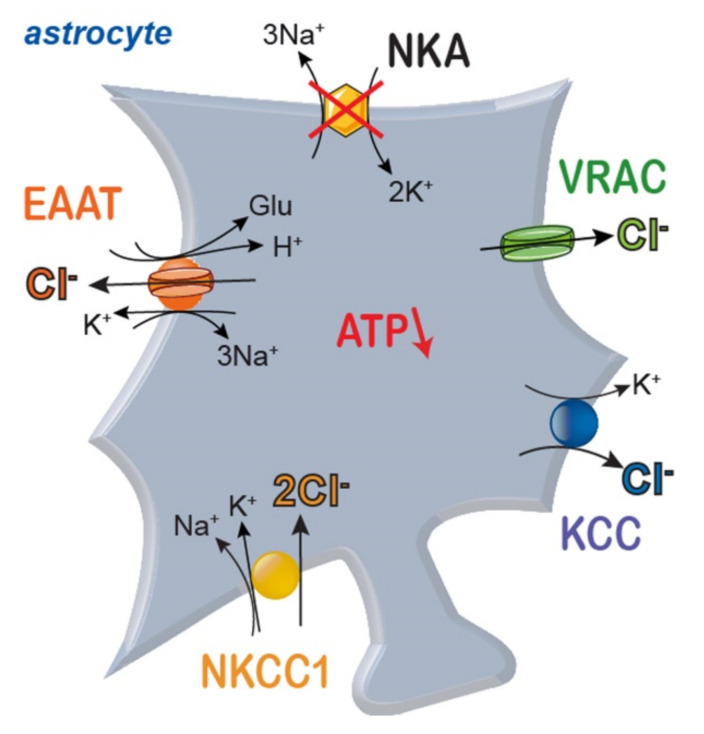
Scheme of main pathways for transport of Cl^−^ across astrocyte membranes. NKA: sodium/potassium-ATPase; NKCC1: sodium-potassium-2 chloride cotransporter 1; EAAT: excitatory amino acid transporter; KCC: potassium-chloride cotransporter; VRAC: volume-activated anion channels.

All (astro-)glial [Cl^−^] determined thus far are clearly above the expectations for passive distributions, and at present, NKCC1 is the only candidate to actively accumulate Cl^−^ (Figure 4). We therefore should expect significantly decreased astrocytic [Cl^−^]_i_ in knock-out animals or in patients carrying mutation in *SLC12A2* (the gene encoding NKCC1) that might impair various brain functions. This was, however, not the case. In *Slc12a2-/-*mice, synaptic density and brain morphology were not profoundly altered [212,213]. There are naturally occurring mutations that abolish NKCC1 expression [214] or impair transport functions [215]. In one of them, reduced cerebral volume and enlarged arachnoid spaces were shown at the age of 9 months [214]. It thus appears from these studies that glial active Cl^−^ accumulation is not absolutely crucial for many brain functions. Alternatively, there might exist additional yet to be identified Cl^−^ accumulating transport processes.

### 4.2. Chloride and Ischemia

All astrocytic anion transport processes are controlled by parameters that are expected to change during brain ischemia. As discussed above, energy restriction will reduce the activity of the NKA and consequently Na^+^ and K^+^ gradients across glial membranes. The resulting membrane depolarization increases ambient glutamate concentration under ischemic stress. The consequences on glial [Cl^−^]_i_ however, can at present not be decisively predicted. There is convincing data that demonstrates NKCC1-mediated Cl^−^ accumulation in astrocytes under ischemic conditions [143,216,217,218,219,220]. The expected ischemia-associated drop in astrocytic [K^+^] would, however, also stimulate Cl^−^ outward transport by KCCs. EAAT anion channels will be stimulated by increased extracellular glutamate, but the astrocytic depolarization might invert Cl^−^ efflux into Cl^−^ influx. Finally, since [Cl^−^]_i_ is in dynamic equilibrium between Cl^−^ accumulation and outward transport/efflux, the consequences of ischemia on glial Cl^−^ homeostasis will also depend on the starting values of [Cl^−^]_i_ in a given cell and preparation.

In astrocytes, stimulation of NKCC1 by increased extracellular K^+^ not only results in accumulation of Cl^−^, but also in glial swelling during ischemia [143,216,217,218,219,220] and in opening of volume-activated anion channels (VRACs or VSOCs, [221,222,223]). These channels permit Cl^−^ efflux and allow regulatory volume decreases together with K^+^ efflux through channels active under resting conditions. Volume-activated anion channels exhibit large pore diameters, and channel activation results in glutamate efflux and excitotoxic tissue damage [224]. Steric and electrostatic interactions with Cl^−^ might result in changes of neurotransmitter efflux upon altered astrocytic [Cl^−^]_i_. Again, for variable astrocytic [Cl^−^]_i_-depending on expression levels of NKCC1, KCCs and EAATs-we expect different degrees of cell swelling and glutamate release.

Under physiological conditions, astrocytes utilize Na^+^ and Cl^−^ gradients for secondary-active GABA [225,226] and glycine uptake [227]. Since GABA and Cl^−^ transport are coupled in a 1:1 stoichiometry, the driving force for GABA uptake will change inversely proportional with [Cl^−^]_i_, i.e., a twofold higher [Cl^−^]_i_ will increase the equilibrium concentration of GABA in the ECS by 100% [228]. Whether such processes play a role in human diseases is currently unclear.

Astroglial Cl^−^ homeostasis thus not only represents a main determinant of volume regulation, but also emerges as an important regulator of synaptic transmission. We are anxiously awaiting a more comprehensive understanding how astrocytes regulate [Cl^−^]_i_, and how changes in these concentrations are compensated under physiological and pathophysiological conditions. We are used to assume that cellular ion concentrations are fixed values without significant variation between different cell types. Cl^−^ appears to be an exception of this concept. There is marked variability in [Cl^−^]_i_ among different tissues, ranging from values only slightly above passive distribution in muscle [229,230] to 150 mM in glioblastoma cells [231]. Future work will clarify whether there is such variability also between different glia cells in distinct brain areas.

## 5. Generation of Cell Swelling and Cerebral Edema

### 5.1. Volume and Water Transport in Animal Cells

The volume of a cell basically reflects the intracellular amount of water [232], and in most animal cells, cell volume is actively regulated [233]. Because animal cells have non-rigid cell walls that limit the generation of a significant hydrostatic pressure but allow passage of water molecules, the intra- and extracellular osmolarity need to be tightly controlled to maintain cell volume [234,235,236]. Cell volume is therefore essentially defined by the total amount of osmolytes [237].

The lipid membrane of cells has a very limited permeability for water [238], but water molecules can also cross the membrane through some transporters [239,240] and ion channels [241,242,243]. In many cells, the transmembrane passage of water is through aquaporin (AQP) channels, mainly expressed in astrocytes [179,244]. Central nervous system neurons presumably lack functional AQP, suggesting that their cell volume is more resistant to changes in extracellular osmolarity. Indeed, neurons do maintain their cell volume if exposed to short periods (20 min) of acute osmotic stress, while astrocytes significantly swell [245]. However, if energy is deprived or extracellular K^+^ concentrations are increased, neurons depolarize and swelling does quickly occur, presumably resulting from opening of non-aquaporin water transport pathways [180], including the EAATs and NKCC1 [246,247]. Differences in the cytoskeletal matrix may also be involved in the differential sensitivity to osmotic stress [232].

**Figure 5:**
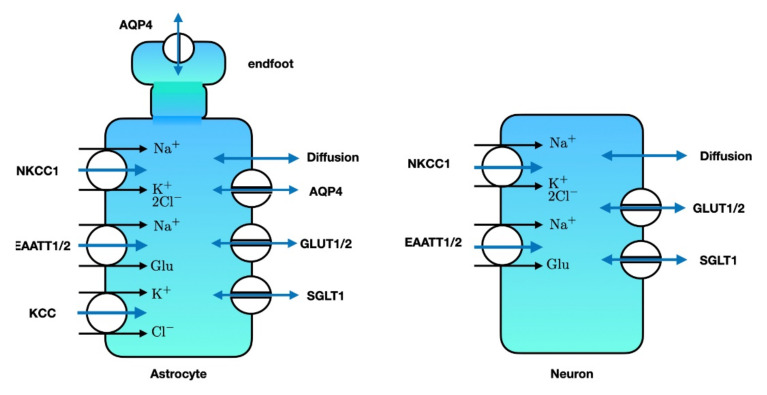
Major transporters and channels that allow water to enter the astrocyte (**left**) or neuron (**right**). Single-headed blue arrows denote water co-transport, while double-headed blue arrows denote passive water transport. SGLT: sodium glucose co-transporter. GLUT: glucose transporter. EAAT: excitatory amino acid transporter. NKCC1: sodium-potassium-2 chloride-co transporter. AQP4: aquaporin-4. KCC: potassium-chloride co-transporter. Note that the astrocytic endfeet mainly contain AQP4 for water transport. Illustration adapted from [178,248].

The major routes for water transport across the membrane of the neuron and astrocyte are shown in Figure 5. Although water is transported via the various energy dependent co-transporters, the main route is via passive transport. The flow of water across the membrane can therefore essentially be described as *filtration*: movement resulting from a hydrostatic and ionic pressure gradient.

In patients with ischemic stroke, significant osmotic imbalances and changes in membrane potential occur, and cell swelling may follow, depending on the depth and duration of the energy depletion [16,235,248]. This increase in cell volume, *cytotoxic* or *cellular edema*, does not change the total brain volume, as fluid is essentially moved from the ECS to the intracellular compartments. In several patients, however, the cytotoxic edema is followed by increased water transport across the blood brain barrier (BBB), increasing the total brain volume, causing *cerebral edema* or brain swelling [248,249]. This process evolves at time scales of hours to days after the primary insult and is a major contributor to secondary brain damage as brain volume is limited by the rigidity of the skull [250]. We will briefly review the fundamental biophysical principles involved in the generation of cell swelling and cerebral edema.

### 5.2. Ion Concentrations and Osmolarity at the Gibbs-Donnan Equilibrium

To understand the direction of ion movement and changes in ion concentrations as a result of energy deprivation, let us assume that all ATP-dependent processes are halted. Cells will then evolve to their thermodynamic equilibrium and each permeant species (Na^+^, K^+^ and Cl^−^) will be in Nernst equilibrium at the same value of the membrane potential, expressed as [251,252]:

(1) $$\frac{{\left[{\mathrm{Na}}^{+}\right]}_{i}}{{\left[{\mathrm{Na}}^{+}\right]}_{e}}=\frac{{\left[K^{+}\right]}_{i}}{{\left[K^{+}\right]}_{e}}=\frac{{\left[{\mathrm{Cl}}^{-}\right]}_{e}}{{\left[{\mathrm{Cl}}^{-}\right]}_{i}}\;$$

resulting in the equilibrium [246]:

(2) $$\left({\left[{\mathrm{Na}}^{+}\right]}_{i}+{\left[K^{+}\right]}_{i}\right){\left[{\mathrm{Cl}}^{-}\right]}_{i}=\left({\left[{\mathrm{Na}}^{+}\right]}_{e}+{\left[K^{+}\right]}_{e}\right){\left[{\mathrm{Cl}}^{-}\right]}_{e\;}\;$$

with [*X*]*_i,e_* the intracellular (*i*) or extracellular (*e*) concentration of ion species *X*. The equilibrium potential at T = 37 °C is given by the Nernst equation [252,253]:

(3) $$V_{\mathrm{gd}}=-61\log_{10}\frac{{\left[X^{+}\right]}_{i}}{{\left[X^{+}\right]}_{e}}\;\mathrm{mV}\;$$

with *X* the concentration of Na^+^, K^+^ or Cl^−^.

Further, the sum of all freely moving charges in a solute is zero (electroneutrality) [253]. The intracellular (and, to a lesser extent the extracellular) fluid also contain non-permeable negatively charged macromolecules, with equivalent concentrations [A^−^]*_i_*, and [A^−^]*_e_*, respectively [251]. For the intracellular fluid this implies:

(4) $${\left[{\mathrm{Na}}^{+}\right]}_{i}+{\left[K^{+}\right]}_{i}={\left[{\mathrm{Cl}}^{-}\right]}_{i}+{\left[A^{-}\right]}_{i}\;$$

and for the extracellular compartment we require:

(5) $${\left[{\mathrm{Na}}^{+}\right]}_{e}+{\left[K^{+}\right]}_{e}={\left[{\mathrm{Cl}}^{-}\right]}_{e}+{\left[A^{-}\right]}_{e}.\;$$

The intracellular Na^+^ at equilibrium can now be expressed as [252]

(6) $${\left[{\mathrm{Na}}^{+}\right]}_{i}={\left[{\mathrm{Na}}^{+}\right]}_{e}\frac{{\left[A^{-}\right]}_{i}+\sqrt{{\left[A^{-}\right]}_{i}^{2}+4\beta \left(\beta -{\left[A^{-}\right]}_{e}\right)}}{2\beta }$$

with *β* = [Na^+^]*_e_* + [K^+^]*_e_*. All ion concentrations at this equilibrium are thus completely defined by the concentrations of extracellular Na^+^ and K^+^ and the equivalent intracellular and extracellular non-permeable anion concentrations.

For example, setting [Na^+^]*_e_* = 140 mM, [K^+^]*_e_* = 15 mM, [A^−^]*_e_* = 0 mM and [*A*^−^] *_i_*= 125 mM, we find that [Na^+^]*_i_* = 207 mM, [K^+^]*_i_* = 22.2 mM, [Cl^−^]*_i_* = 105 mM, [Cl^−^]*_e_* = 155 mM and *V*_gd_ = −10.4 mV. The non-zero membrane potential is known as the Gibbs-Donnan potential that results from the difference in the equivalent concentrations of the intra- and extracellular impermeant ions (cf Equation 6); if the difference would be zero, all ion gradients at equilibrium vanish with corresponding membrane potential *V_m_* = 0 mV.

We can also estimate the osmotic pressure at this Gibbs-Donnan equilibrium by considering the concentration difference of the non-organic ions, only, and neglect the (much smaller) contribution of the charged organic macromolecules and other, non-charged, solutes. Using the numerical example above, ∆*c* ≈ 24.2 mM, resulting in an osmotic pressure of *π* = RT ∆*c =* 6 × 10^4^ Pa. As the hydrostatic pressure that neurons and astrocytes can generate (approximately 300 Pa [251]) is much smaller than the osmotic pressure, the water flux is essentially defined by the osmotic pressure difference [248,251,254].

The presence of both a low intracellular osmolarity and low intracellular Na^+^ in living cells implies that they operate far from their thermodynamic equilibrium. The main mechanism that prevents accumulation of intracellular Na^+^ and-at the same time- maintains a low intracellular osmolarity, is the NKA in the neuronal and astrocytic cell membrane, in combination with their low Na^+^ permeability at the resting membrane potential [246,248,251].

### 5.3. Dynamics of Cell Volume Changes during Metabolic Stress

During energy deprivation, the number of ions in the extra- and intracellular space will not be constant as cells will accumulate Na^+^ and excrete K^+^ as they will tend towards the Gibbs-Donnan equilibrium. The increase in extracellular K^+^ cannot be fully compensated by astrocytes [103,255,256,257], as their buffering capacity is limited and compromised during metabolic stress [258] and membrane potentials will decrease. Depending on the depth and duration of the energy deprivation, the excess intracellular Na^+^ is not always compensated with a similar efflux of K^+^ to maintain electroneutrality [235,254] and Cl^−^ influx is needed to maintain electroneutrality. This results in an increase in intracellular osmolarity, and cell swelling is inevitable [235,248,254,259,260].

How fast neurons or astrocytes will swell is a function of the effective water permeability. Astrocytes can very quickly change their volume in response to changes in osmolarity, mainly at their perivascular endfeet where the AQP4 density is largest [244,261,262]. For neurons, the permeability for water in physiological conditions is much smaller, and volume changes in response to changes in osmolarity are slower (Andrew et al., 2007). Water permeability can also change by e.g., differences in the activity of NKCC1 [180].

Biophysical models can also further our understanding of the complex interactions between the several processes involved in ion and volume homeostasis [254,257]. A detailed model of the tripartite synapse confirms the essential role of glia to maintain ion gradients and cell volume for reliable neurotransmission and also elucidates the relative importance of several energy dependent processes involved in the development cytotoxic edema at different levels and durations of metabolic stress [258].

### 5.4. Cerebral Edema

**Figure 6:**
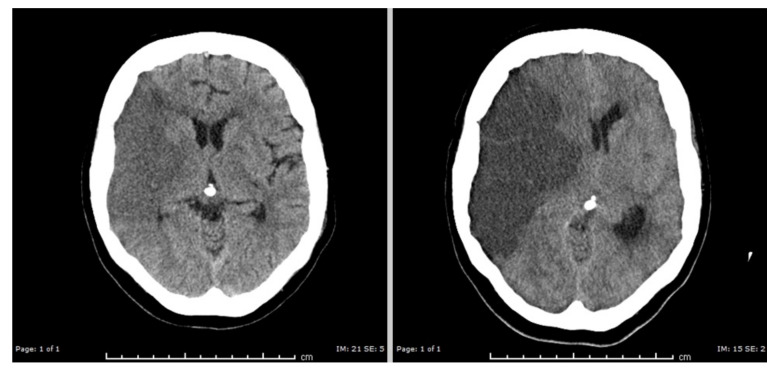
Cerebral edema resulting from an ischemic stroke. Left: head CT with hypodense gray and white matter on the right side of the brain (left in the picture). This essentially reflects cytotoxic cell swelling in the acute phase of the infarction. Right: head CT two days later showing massive cerebral edema of the infarcted tissue, with displacement of brain tissue to the left side. Courtesy of M. Hazewinkel, radiologist Medisch Spectrum Twente. “Reused with permission of Springer, (c) MJAM van Putten [252]”.

While cell swelling (cytotoxic edema) expresses redistribution of extracellular water into the intracellular compartment, a net flux of water through the blood brain barrier (BBB) generates cerebral edema [248,249,263,264]. This most often results from severe traumatic brain injury, hypoxia or ischemia (Figure 6).

**Figure 7:**
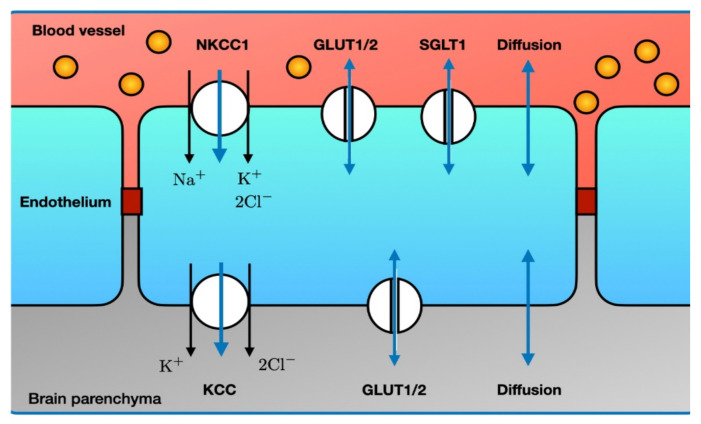
Major routes for influx of water through the blood brain barrier. Single-headed blue arrows denote water co-transport, while double-headed blue arrows denote passive water transport. Note the asymmetry in the various transporters between the luminal and abluminal side. Endothelial tight junctions (dark red) prevent leakage of macromolecules (e.g., albumin, yellow circles) from the capillary blood. The many other ion transporters and ion channels that do not transport water are not shown. Abbreviations: see text. Illustration adapted from [248].

The BBB is a dedicated barrier between the arterial blood flow to the brain and the brain parenchyma, created by functionally asymmetric endothelial cells (Figure 7). At the luminal side, the endothelial cells contain NKCC1, GLUT1/2 and SGLT1. At the abluminal side, the main transporter that co-transports water is KCC. Both endothelial sides also allow water to enter through diffusion.

The first phase of water flux through the BBB results from an increase in the Na^+^ gradient between the capillary blood and the ECS: *ionic edema*, mediated by the endothelial ion channels and transporters. At this stage, the integrity of the BBB is structurally intact [263]. *Vasogenic edema* results from a disruption in the BBB and extravasation of serum proteins (such as albumin) into the cerebral parenchyma [248,264,265,266]. These serum proteins will add to the osmotic pressure gradients across the BBB, furthering water movement into the brain parenchyma. In pioneering work by Ito et al. it was shown that in experimental stroke in Mongolian Gerbils cytotoxic edema developed nearly immediately. The onset of vasogenic edema, measured by passage of ^131^I-albumin from blood into the brain, occurred after restoration of blood flow, with peak values at about 20 h [263]. Return to baseline varied with a maximum duration up to 1 week. Such timescales are also observed in the clinic [250,267]. A major risk resulting from cerebral edema is compression of otherwise healthy brain tissue, resulting in direct mechanical damage and compression of vasculature, causing secondary ischemia (Figure 7).

During disease states, transporter expression can change. For example, total NKCC activity in endothelial cells was reduced in an experimental stroke model [268] and GLUT1 and SGLT1 activity were increased during OGD in endothelial cells co-cultured with astrocytes [269]. It is also reported that NKCC1 activity can increase in stroke, contributing to more excretion of Na^+^, Cl^−^ and water across the BBB [270,271]. In mice exposed to permanent focal ischemia, activity of SGLT1 and GLUT1 was increased and inhibition of SGLT1 with phlorizin decreased infarct and edema [269].

## 6. Conclusions

The astrocyte is a multitalented cell. Its functions are essential for Na^+^, K^+^, and Cl^−^ homeostasis, control of pH and cell volume which are key to action potential generation and propagation and for reliable neurotransmission at the tripartite synapse. Astrocytic dysfunction, either in isolation or during energy depletion, results in significant reversible and irreversible changes in neural functioning synaptic transmission and water homeostasis. A better understanding of the various (energy dependent) astrocytic functions holds large potential to add to identification of novel, targeted treatments to improve recovery of patients with ischemic stroke. This includes targets to limit irreversible cell damage and indirect damage resulting from cerebral edema.
